# Evaluation of Pediatric COVID-19 Screening Process in a Tertiary Hospital of Indonesia

**DOI:** 10.1155/2022/6194776

**Published:** 2022-04-20

**Authors:** Nastiti Kaswandani, Nina Dwi Putri, Madeleine Ramdhani Jasin, Tartila Tartila, Henny Adriani Puspitasari, Wahyuni Indawati, Mulya Rahma Karyanti, Ari Prayitno, Niken Wahyu Puspaningtyas, Yogi Prawira, Ajeng Kartika Ayu Putri, Hana Anindya Indana, Aryono Hendarto

**Affiliations:** Department of Pediatrics, Cipto Mangunkusumo National Central Hospital, Faculty of Medicine, Universitas Indonesia, Jakarta, Indonesia

## Abstract

**Objectives:**

To identify parameters that can improve the effectiveness of COVID-19 screening in the pediatric population according to the demographic, clinical, and epidemiological characteristics of pediatric patients screened for COVID-19 at our hospital.

**Methods:**

A cross-sectional study of suspected and confirmed pediatric patients (0–18 years old) with COVID-19 using data from the electronic medical records of Dr. Cipto Mangunkusumo Central Hospital from March to December 2020.

**Results:**

From 1,018 data of suspected COVID-19 pediatric patients, there were 94 (9.2%) confirmed cases of COVID-19. The proportions of children with travel history (*p*=0.022), positive contact history (*p* < 0.001), fever ≥38°C (*p*=0.034), cough (*p*=0.038), and abdominal pain (*p*=0.022) were significantly higher in the confirmed COVID-19 group compared to the non-COVID-19 group.

**Conclusions:**

A majority of the confirmed COVID-19 pediatric patients have travel and positive contact history, along with symptoms of fever, cough, and abdominal pain. However, these are nonspecific symptoms that may also be misdiagnosed as other diseases. Improving access and turnaround time of the RT-PCR test is mandatory, as no specific screening variables have been identified.

## 1. Introduction

Coronavirus disease-2019 (COVID-19) has emerged as a significant public emergency since its declaration as a global pandemic by the World Health Organization (WHO) in March 2020, causing numerous deaths and massive economic catastrophes across the globe [1, 2]. While epidemiological data show that COVID-19 in children is not as common as in adults, the incidence of pediatric cases in Indonesia has accelerated [3]. The clinical manifestations of COVID-19 in pediatric patients are variable and range from asymptomatic to acute upper respiratory tract infection, gastrointestinal symptoms with shock, as well as coagulation disorders in severe cases [4]. The risk of severe disease tends to be higher in infants and children with comorbidities or underlying conditions [5]. Existing studies have shown that pediatric patients tend to develop asymptomatic to mild diseases and are less likely to require intensive care or other adjuvant therapies that are indicated for those with severe clinical manifestations [6, 7].

The clinical presentation and epidemiological characteristics of severe acute respiratory syndrome coronavirus-2 (SARS-CoV-2) are variable and relatively unclear. It is challenging to diagnose COVID-19 based on its clinical presentation, especially as Indonesia has a high burden of major infectious and tropical diseases, such as malaria and dengue fever [8]. Therefore, confirming the diagnosis of COVID-19 depends on a positive real-time polymerase chain reaction (RT-PCR) or antigen testing result. Unfortunately, the results of the RT-PCR test may require multiple days to be released, which corresponds to a longer length of stay for the patient as well as a delay in proper medical treatment. Patients who live outside the capital region with limited resources may have to wait even longer for these results compared to those in the capital region [9, 10]. Laboratory results in pediatric COVID-19 cases have shown lower hemoglobin levels and higher white blood cell counts in some studies, although other abnormal laboratory findings are not specific to this disease [3, 11]. These diagnostic limitations in clinical patterns may lead to a challenge in the early recognition of COVID-19 in children [12].

In most hospitals in Indonesia, implementing COVID-19 screening in the pediatric population is problematic due to the unspecific signs and symptoms and limited immediate access to SARS-CoV-2 RT-PCR results. Balancing early diagnosis, infection control, and lack of isolation facilities also remains a huge challenge. Therefore, this study aims to identify the parameters that can improve the effectiveness of COVID-19 screening in the pediatric population based on an evaluation of the demographic, clinical, and epidemiological characteristics of pediatric patients screened for COVID-19 at our hospital.

## 2. Methods

### 2.1. Patients and Clinical Data

**Figure 1:**
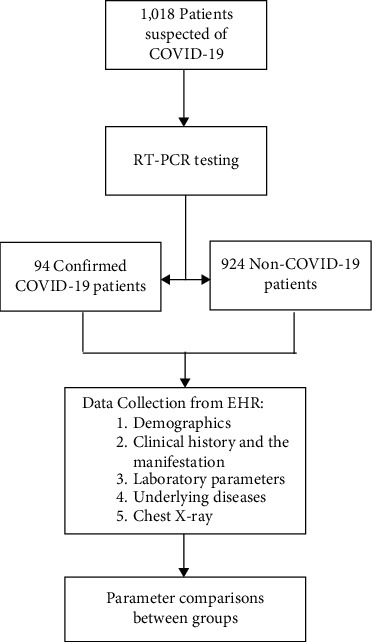
Study flow.

We performed a cross-sectional study of suspected and confirmed pediatric patients with COVID-19 using the electronic medical records of Dr. Cipto Mangunkusumo Central Hospital, a national referral hospital in Indonesia, from March 2020 to December 2020 (Figure 1). Based on WHO guidelines, our hospital screened all patients admitted to the emergency room and clinic during the pandemic. Patients with clinical symptoms consistent with suspected or probable COVID-19 diagnostic criteria were admitted to the isolation ward. Nasopharyngeal and oropharyngeal swabs were performed at the emergency room or clinic by trained physicians, and were subsequently delivered to the laboratory to be examined for RT-PCR of SARS-CoV-2. A diagnosis of COVID-19 was established following a positive RT-PCR test. In the event of uncertainty in the diagnosis, an expert in new-emerging and re-emerging diseases was consulted to determine whether the clinical presentation of the patient is consistent for suspected or probable COVID-19 criteria.

All pediatric patients (0–18 years old) included in this study were diagnosed based on the following clinical criteria from the WHO guidelines: (1) acute onset of fever and cough or (2) acute onset of any three or more of the following symptoms: fever, cough, fatigue, headache, myalgia, sore throat, coryza, dyspnea, nausea/vomiting, diarrhea, and altered mental status. Epidemiological criteria for diagnosis included: (1) residing or working in an area with a high risk of viral transmission of or closed residential settings any time within 14 days prior to symptom onset; (2) residing or traveling to an area with community transmission anytime within the 14 days before symptom onset; or (3) working in any healthcare setting within 14 days before symptom onset [13]. After applying the diagnostic criteria, the diagnosis of COVID-19 was confirmed using the RT-PCR test. For each patient, at least two consecutive RT-PCR tests were performed to increase the sensitivity. Patients who tested positive in the RT-PCR test were classified into the confirmed COVID-19 group (group A), and patients who tested negative were classified into the non-COVID-19 group (group B). In this study, travel history was defined as residing or traveling to an area with community transmission any time within 14 days prior to symptom onset, and contact history was defined as direct contact or being in a proximity of within 1 metre for at least 15 minutes with a confirmed COVID-19 patient or previous exposure to a healthcare facility.

### 2.2. Pediatric Screening Flow

In our hospital, patient admission can be accessed through the emergency unit or outpatient care by three different procedures: (1) a direct visit without an appointment, with patients screened at triage; (2) referral from other health care facilities, with patients previously classified as suspected or confirmed COVID-19 cases directed to the COVID-19 triage, and non-COVID-19 patients directed to the non-COVID-19 triage; and (3) online registration, in which patients complete a self-evaluation form before admission to determine whether the patient will be directed to the COVID-19 or non-COVID-19 triage when first admitted.

**Figure 2:**
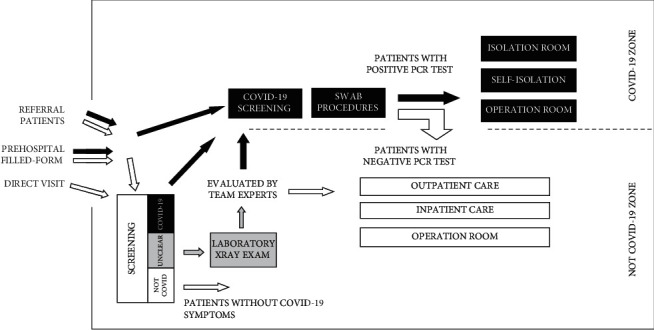
The screening process for COVID-19 in pediatric patients at our facility.

The initial screening procedure is completed by evaluating the clinical characteristics of the patients as well as epidemiological criteria from the WHO guidelines to determine whether the patient is suspected to have COVID-19. Patients who meet the criteria are categorized as suspected COVID-19 patients and directed to COVID-19 screening. Patients with unclear characteristics are categorized as undetermined patients and are required to complete laboratory or X-ray procedures, which are then further analyzed by an expert team to determine whether the patient is classified as a suspected COVID-19 case. After screening, patients with suspected or probable COVID-19 criteria undergo an RT-PCR examination to confirm the diagnosis of COVID-19 (Figure 2).

### 2.3. Statistical Analysis

Statistical differences between groups were analyzed using the bivariate independent *t*-test or the Mann−Whitney test. Categorical variables were analyzed using the *χ*^2^ or Fisher's exact test. Differences were considered statistically significant if the *p*value was <0.05. The data analysis was performed using Statistical IBM SPSS Statistics for Windows, version 26.0 64 bit (IBM SPSS Statistics, New York, US).

## 3. Results

### 3.1. Demographics

During the study period, 1,018 subjects were diagnosed as suspected COVID-19 patients, with 94 subjects (9.2%) subsequently confirmed as COVID-19-positive by RT-PCR. Male subjects dominated both the confirmed (57.4%) and non-COVID-19 (55.1%) groups (Table 1). However, there were no significant differences in the sex distribution between the two groups (*p*=0.661). Children with confirmed COVID-19 had a higher median age than those in the non-COVID-19 group (101 months versus 21.5 months, *p* < 0.001). The proportion of children > 10 years of age was higher in the confirmed COVID-19 group than in the non-COVID-19 group (42.6% versus 22.0%; *p* < 0.001).

### 3.2. History and Clinical Manifestations

More than half of the patients with confirmed COVID-19 had a travel history (70.3%; *p* < 0.001) within 14 days and a positive contact history (51.1%; *p* < 0.001) with other confirmed COVID-19 patients. Patients with confirmed COVID-19 had higher frequencies of certain symptoms than non-COVID-19 patients, including fever ≥38°C (65.6% versus 54.1%; *p*=0.034), cough (45.2% versus 34.4%; *p*=0.038), and abdominal pain (15.1% vs 8.0%; *p*=0.022), with fever having the highest incidence (65.6%) compared to other symptoms. Other symptoms, such as dyspnea, runny nose, diarrhea, vomiting, skin rash, and shock, were not significantly different between the confirmed and non-COVID-19 subjects (Table 1).

## 4. Discussion

From the 1,018 pediatric patients with suspected COVID-19, 94 were confirmed to be positive, while the remaining 924 tested negative for COVID-19 following RT-PCR examination. This proportion is lower than that documented by a study in Mexico [14], which reported 15.9% confirmed cases from 1,849 children analyzed. A possible reason for this discrepancy is that this study was performed in a referral hospital, with a majority of patients arriving with various complaints due to their underlying diseases. This study found a higher proportion of children aged >10 years old in the confirmed COVID-19 group compared to that in the non-COVID-19 group. This is consistent with a previous cross-sectional observational study by Noman et al., which reported that 13.8% of the children diagnosed with COVID-19 were <1 year old, 20.7% was between 1–5 years old, 24.1% were between 6–10 years old, and 41.4% were between 11–15 years old [15]. The high mobility of children who are older than 10 years old, which can be similar to that of young adults and adults, is thought to contribute to their higher percentage of confirmed COVID-19 cases. In a case-control study, close contact with persons infected with COVID-19, such as household members, visitors at home, and attendees of gatherings outside the household (including social functions or activities with other children), was also associated with SARS-CoV-2 infection in adolescents [16].

Travel and positive contact history have a notable relationship with COVID-19. Numerous studies have indicated that most pediatric patients with COVID-19 have a history of traveling to epidemic regions, while some have a history of close contact with other confirmed COVID-19 patients [17, 18]. A study of 24 confirmed COVID-19 children in Taiwan reported that 20 patients (83.8%) had a travel history, while the remaining 4 had a definite direct or indirect contact history with someone who had travelled to another country in the recent past [3]. Therefore, preventive procedures such as use of masks or face shields, physical distancing, and hand hygiene procedures still remain as the most important aspects in preventing the transmission of COVID-19. However, there are also cases of pediatric COVID-19 patients with no apparent history of exposure. Therefore, even when there is no definite epidemiological history, it is suggested that COVID-19 should not be excluded entirely in children, especially those who live in a region with high positivity rates such as Indonesia, and this must be comprehensively evaluated by taking into consideration other symptoms or examination results. This study found that the most common symptoms were fever, followed by non-specific symptoms of the upper respiratory tract such as cough, as well as abdominal pain. Other studies have also shown that children with COVID-19 present with a fever and dry cough as the most common clinical manifestations [17–19]. A systematic review reported the most prevalent COVID-19 symptoms in children were fever (47.5%), cough (41.5%), nasal symptoms (11.2%), diarrhea (8.1%), and nausea/vomiting (7.1%) [20]. Greater upper airway resistance in children causes aerosol particles to be more easily deposited in the tracheobronchial tree as opposed to the alveoli, which leads to more bronchiolitis-like infections compared to pneumonia in children with SARS-CoV-2 [21]. Diarrhea may also appear as a symptom since the viral receptor of angiotensin converting enzyme 2 (ACE-2) was found in the epithelial cells of the small intestine, indicating that SARS-CoV-2 can actively infect and replicate in the GI tract [22]. Abdominal pain was also a notable finding suggestive for COVID-19, and this symptom was mainly found in those presenting with diarrhea. Another study also reported that SARS-CoV-2 RNA was still detected in rectal swabs even after seroconversion of nasopharyngeal swab test results [23]. Determining the possible differential diagnosis can therefore be challenging, particularly in an endemic country with various tropical infectious diseases and common viral infections.

### 4.1. Limitations

This study was conducted in a tertiary referral hospital during the first wave of the pandemic in Indonesia. This setting may not be representative of the situation found in primary health care services due to the abundance of patients with underlying diseases and comorbidities, which may lead to biases. Further studies are warranted to analyze COVID-19 patients in primary health care settings with no underlying diseases and comorbidities.

### 4.2. Conclusion

Our study revealed that travel history and positive contact history are important screening questions during COVID-19 triage. Most of the patients presented to the hospital with nonspecific symptoms such as fever, cough, and abdominal pain, which could be misdiagnosed as other infectious diseases, particularly given the tropical country setting. Laboratory parameters, such as neutrophil and D-dimer levels, are not specific for COVID-19, and this therefore limits their usability in the screening process. Improving access and turnaround time of RT-PCR testing is mandatory, as no accurate screening variables have been found. Further screening methods are required to effectively screen and manage COVID-19 in children.

## Figures and Tables

**Table 1:** Clinical characteristics of confirmed and non-COVID-19 subjects.

Parameters	*N*	Confirmed COVID-19 patients *n* (%)	Non-COVID-19 patients *n* (%)	*p* value
Total patients	1018	94 (9.2)	924 (90.8)	
Male	563	54 (57.4)	509 (55.1)	0.661
Female	455	40 (42.6)	415 (44.9)	
Age median (min; max), months	122.5	101 (0; 216)	21.5 (0; 215)	<0.001^*∗*^
Age				
0–5 years	649	41 (43.6)	608 (65.8)	<0.001^*∗*^
5–10 years	126	13 (13.8)	113 (12.2)	
>10 years	243	40 (42.6)	203 (22.0)	

*History and clinical manifestations*				
Travel history	967	64 (70.3)	157 (17.9)	<0.001^*∗*^
Contact history	937	46 (51.1)	171 (20.2)	<0.001^*∗*^
Fever ≥ 38°C	1006	61 (65.6)	494 (54.1)	0.034^*∗*^
Dyspnea	1007	43 (46.2)	387 (42.3)	0.469
Cough	1007	42 (45.2)	314 (34.4)	0.038^*∗*^
Runny nose/congestion	999	14 (15.1)	84 (9.3)	0.074
Diarrhea	1003	25 (26.9)	205 (22.5)	0.341
Nausea and vomiting	1003	31 (33.3)	256 (28.1)	0.290
Abdominal pain	1003	14 (15.1)	73 (8.0)	0.022^*∗*^
Skin rash	1004	9 (9.7)	65 (7.1)	0.371
Shock	1018	22 (23.4)	220 (23.8)	0.930

## Data Availability

No data were used to support this study.
